# Right-Atrial Muscular Ridge as an Isthmus for Macro-Re-Entrant Atrial Tachycardia in Partial Atrial Standstill

**DOI:** 10.1016/j.jaccas.2026.109429

**Published:** 2026-07-23

**Authors:** Ken Yamazaki, Takahiko Kinjo, Masaomi Kimura, Yuji Ishida, Hirofumi Tomita

**Affiliations:** a Department of Cardiology and Nephrology, Hirosaki University Graduate School of Medicine, Hirosaki, Japan; b Department of Advanced Management of Cardiac Arrhythmia, Hirosaki University Graduate School of Medicine, Hirosaki, Japan

**Keywords:** ablation, atrial tachycardia, cardiomyopathy, electrophysiology, tachycardia

## Abstract

**Background:**

Partial atrial standstill is a rare disorder with extensive atrial electrical silence that can coexist with atrial tachycardia (AT). However, the mechanism of AT in this population remains unclear.

**Case Summary:**

A 54-year-old man was referred for AT and complete atrioventricular block. High-resolution electroanatomic mapping revealed extensive electrically silent areas involving the sinus venarum and right atrial septum, consistent with partial atrial standstill. In contrast, a preserved right atrial muscular ridge (arcuate ridge and crista terminalis) formed the critical isthmus of a macro-re-entrant circuit. Ablation of this ridge, followed by cavotricuspid isthmus ablation, terminated the AT. A permanent pacemaker was subsequently implanted. The patient remained free of atrial tachyarrhythmia for 7 years.

**Discussion:**

Preserved muscular ridges can be key conduction pathways and ablation targets in an extensively scarred atrium.

**Take-Home Messages:**

Muscular ridges can serve as a conduction pathway of macro-re-entrant AT in an extensively scarred atrium.

## Visual Summary

**Figure:**
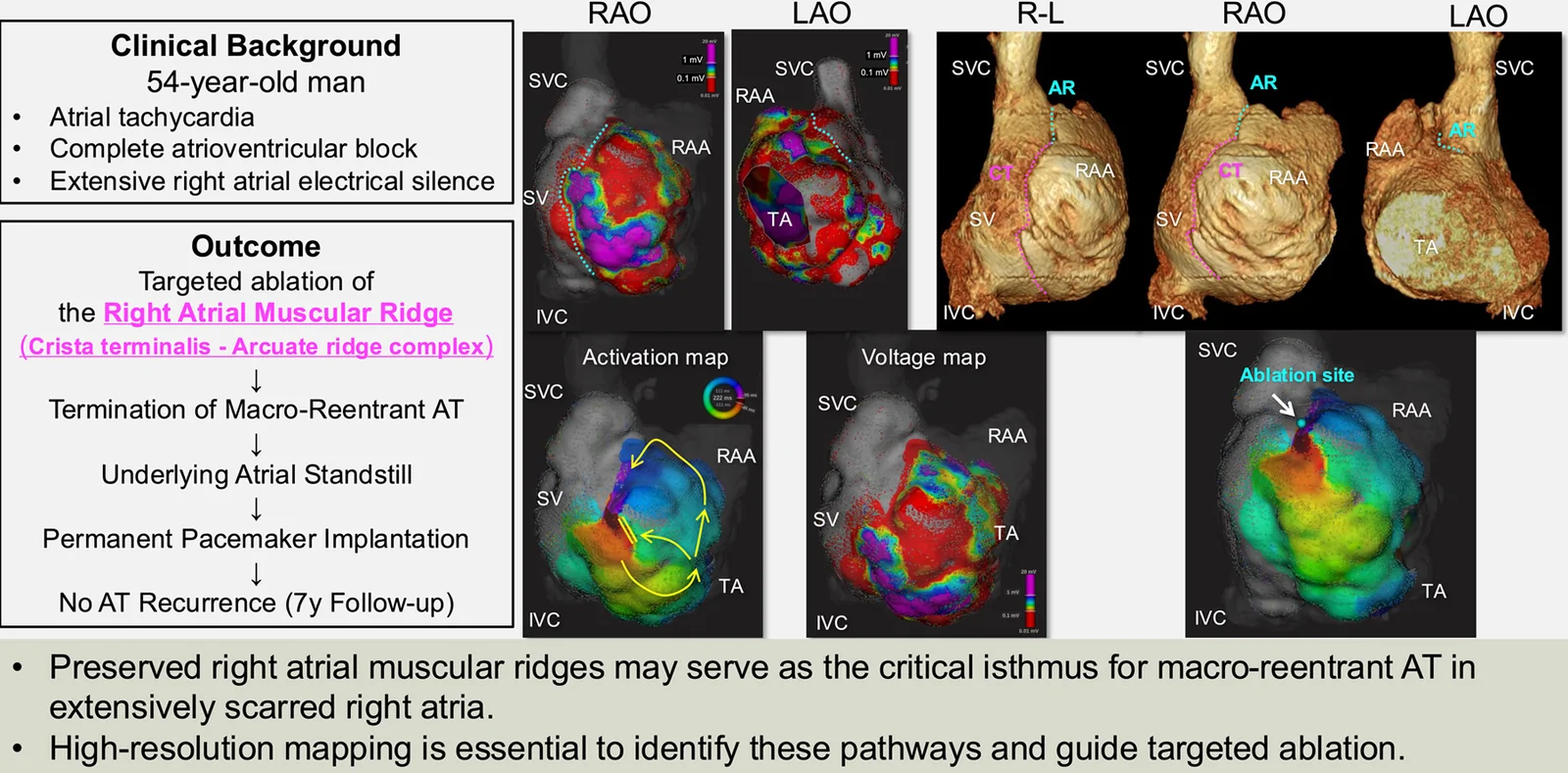

**Visual Summary:**
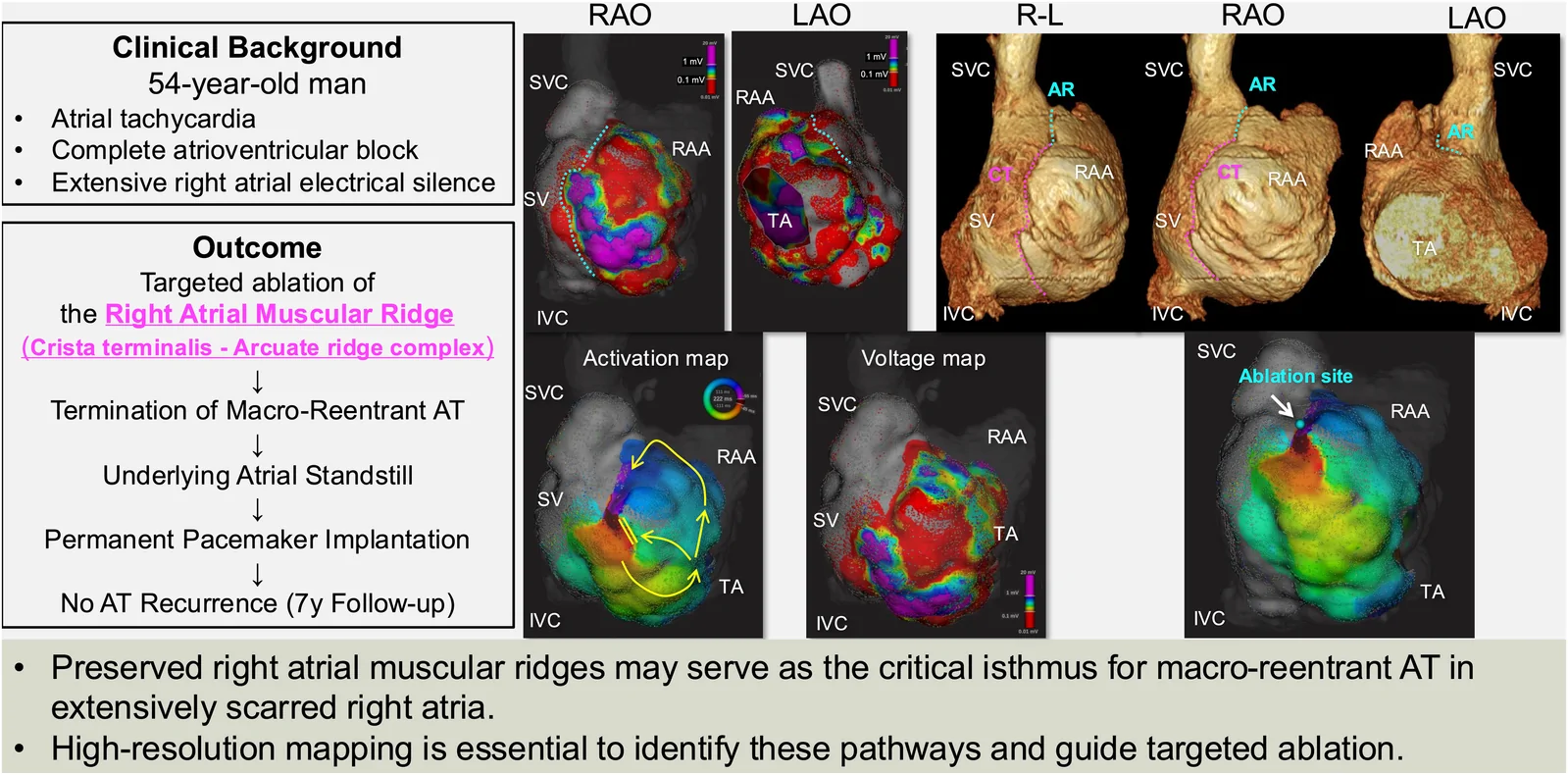
A Continuum of the Right Atrial Muscular Ridge, Including the Arcuate Ridge and the Crista Terminalis, Can Serve as a Critical Isthmus for Macro-Re-Entrant Atrial Tachycardia in a Patient With Partial Atrial Standstill Recognizing this infrequently reported structure and its anisotropic conduction properties is essential for electrophysiologists. AR = arcuate ridge; AT = atrial tachycardia; CT = crista terminalis; IVC = inferior vena cava; LAO = left anterior oblique; RAA = right atrial appendage; RAO = right anterior oblique; R-L = right-to-left view; SV = sinus venarum; SVC = superior vena cava; TA = tricuspid annulus.

Partial atrial standstill is a rare disease characterized by the absence of electrical and mechanical activity in part of the atrium. Previous studies suggest that it may be a progressive condition, leading to atrial fibrillation, atrial tachycardia (AT), and finally, to total atrial standstill.^1^ However, little is known about the etiology and mechanisms of AT in patients with atrial standstill. Herein, we report a case of partial atrial standstill with macro-re-entrant AT, in which a continuum of the right atrial muscular ridge—a series of interconnected myocardial bundles extending from the arcuate ridge to the crista terminalis—served as the critical isthmus.

**Take-Home Messages**

• In patients with extensive right atrial scarring, preserved right atrial muscular ridges may represent the only viable conduction pathway and the critical isthmus for macro-re-entrant atrial tachycardia.
• Systematic high-resolution mapping of these structures is essential to delineate the arrhythmia circuit and guide targeted ablation.

## History of Presentation

**Figure 1:**
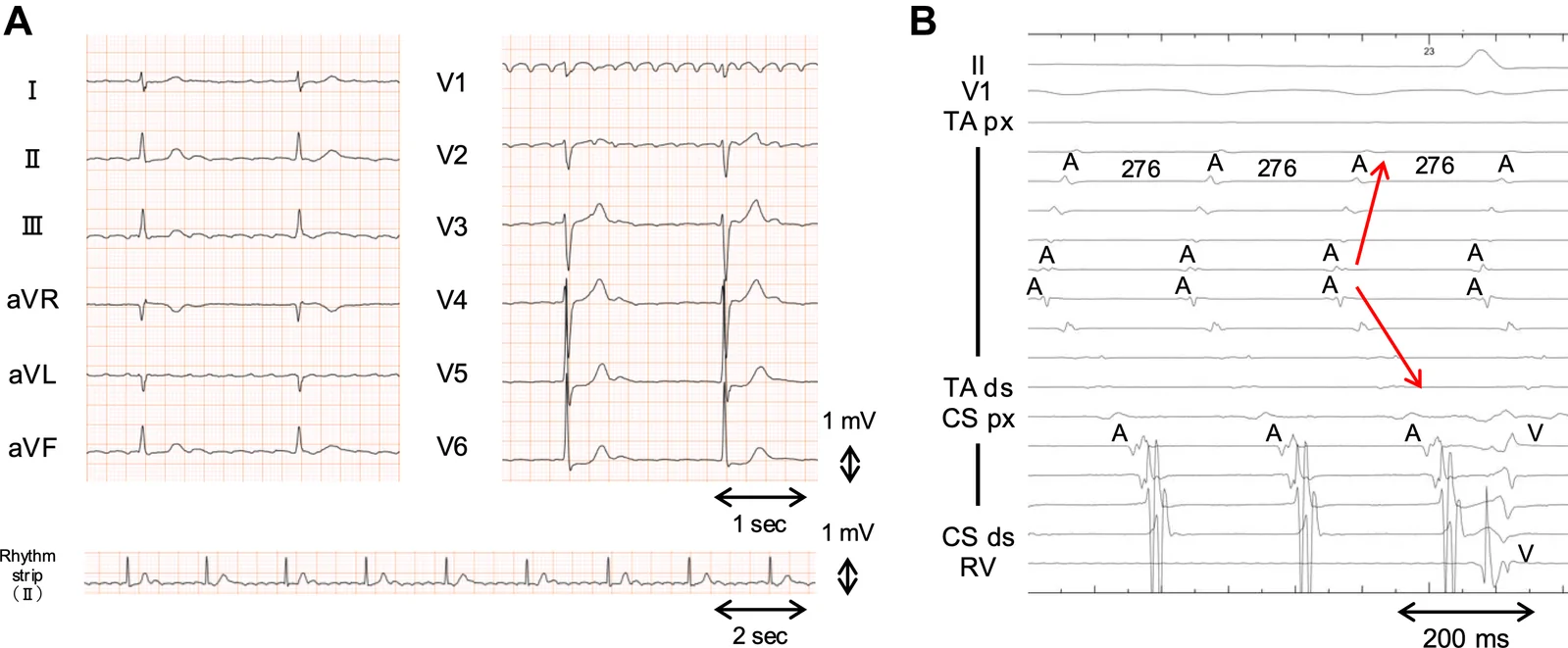
Electrocardiography and Intracardiac Electrogram (A) Twelve-lead electrocardiography showing atrial tachycardia with complete atrioventricular block. (B) Intracardiac electrocardiography demonstrating atrial tachycardia. The arrows indicate the sequence of atrial activation. CS = coronary sinus; ds = distal; px = proximal; RV = right ventricle; TA = tricuspid annulus.

A 54-year-old man was referred to our hospital after asymptomatic AT and complete atrioventricular block were incidentally detected on electrocardiography (Figure 1A). He denied chest pain, dyspnea, palpitations, syncope, or presyncope. On examination, his blood pressure was stable, and bradycardia was noted, with a heart rate of 37 beats/min. Cardiac auscultation revealed no murmurs, and there were no signs of heart failure.

## Past Medical History

The patient had no history of cardiac surgery, congenital heart disease, myocarditis, or systemic inflammatory disease. There was no family history of cardiomyopathy or conduction disorders. He was not taking any medications before presentation.

## Differential Diagnosis

The differential diagnosis included idiopathic atrial standstill, cardiac sarcoidosis, inflammatory or infiltrative cardiomyopathy, atrial scarring related to previous surgery or ablation, and genetic atrial myopathy.

## Investigations

Coronary angiography excluded coronary artery disease and cardiac magnetic resonance imaging demonstrated only mild cardiac enlargement, without late gadolinium enhancement or features suggestive of cardiac sarcoidosis. There was no evidence of extracardiac sarcoidosis.

## Management

The patient was admitted to our hospital for catheter ablation and pacemaker implantation after starting rivaroxaban (15 mg/d).

**Figure 2:**
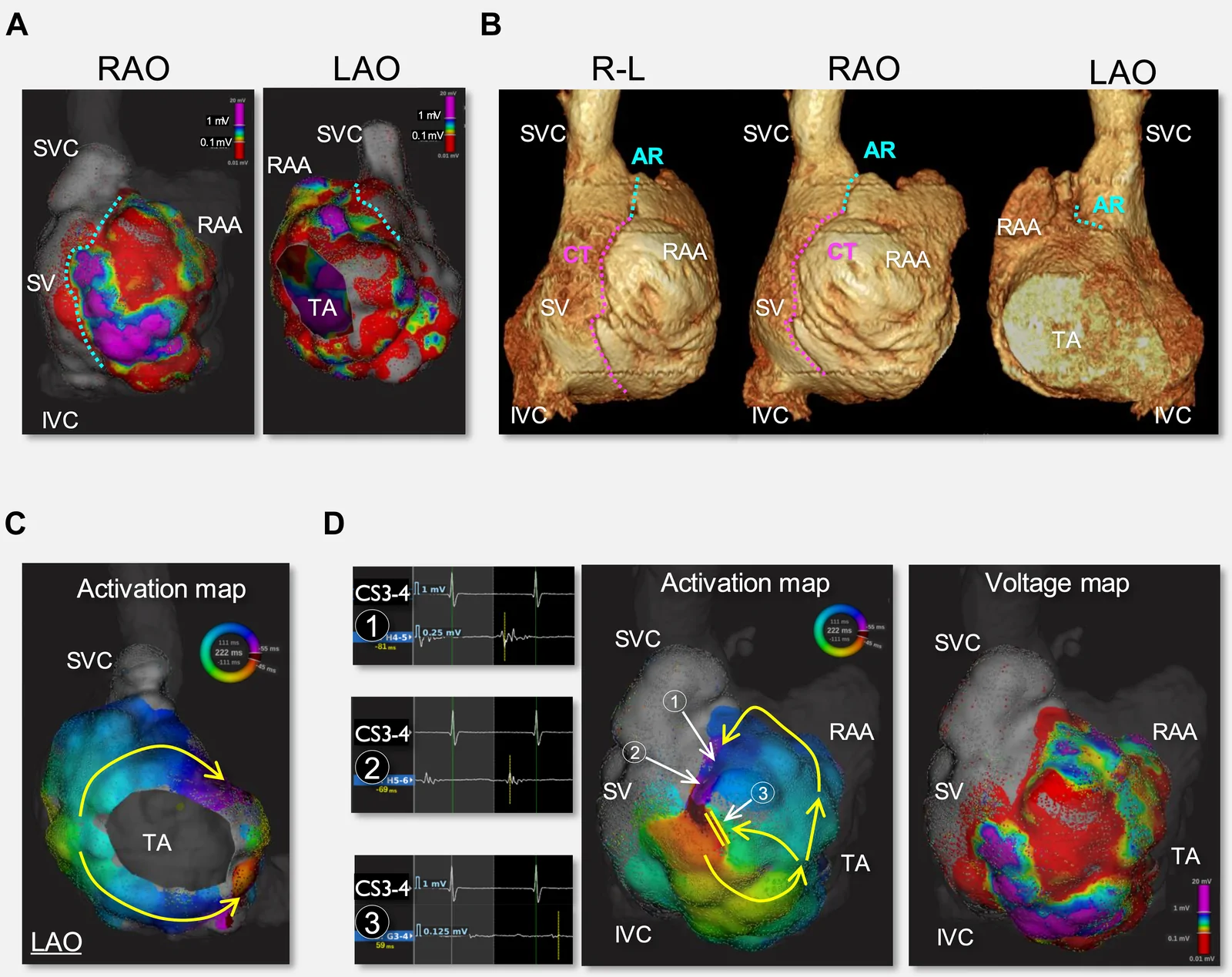
Electroanatomical Mapping and 3-Dimensional Computed Tomography Images (A) Voltage map of the right atrium during atrial tachycardia. Extensive low-voltage areas are observed in the sinus venarum (SV) and right atrial appendage (RAA). A confidence mask cutoff of 0.02 mV was applied during voltage mapping. The continuum of right atrial muscular ridge, encompassing an arcuate ridge (AR) (purple dashed lines) and the crista terminalis (CT) (cyan dashed lines), separates the SV and RAA. (B). Three-dimensional computed tomography images. The right atrial muscular ridge (cyan dashed lines) separates the SV and RAA. (C). Activation map viewed from the left anterior oblique (LAO). (D) Modified right anterior oblique (RAO). Fractionated electrograms are observed at sites corresponding to the AR (site 1) and the CT (site 2). Site 3 shows double potentials reflecting conduction block in the anteroposterior direction. Yellow arrows indicate the direction of wavefront propagation. HRA = high right atrium; IVC = inferior vena cava; R-L = right-to-left; SVC = superior vena cava; other abbreviations as in Figure 1.

**Figure 3:**
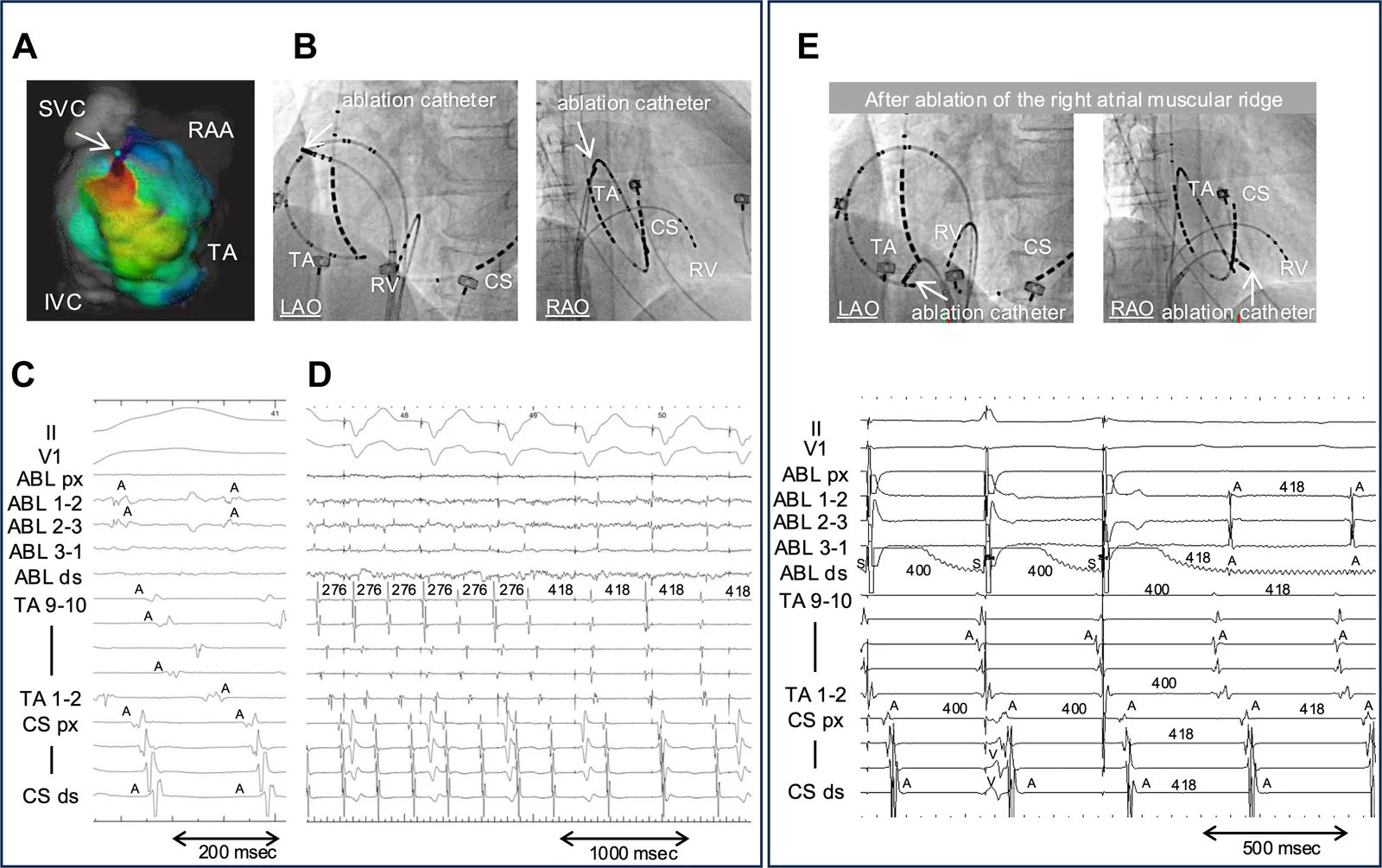
Successful Ablation Site (A) The ablation sites (white arrow) correspond to the transition of arcuate ridge to CT, which is the boundary between the SVC and the RAA. (B) Successful ablation site of the right atrial muscular ridge. (C) Electrograms recorded using the IntellaNav MiFi catheter (Open-irrigated ablation catheter, Boston Scientific). A fractionated electrogram is observed on the microelectrode pairs, whereas no signals are detected on the distal bipolar. (D) Intracardiac electrograms during ablation. After ablation, the activation sequence on the TA changes, and the cycle length is prolonged. (E) After the tachycardia changed, entrainment pacing from CTI confirmed CTI-dependent atrial tachycardia. Abbreviations as in Figures 1 and 2.

The electrophysiological study (Figure 1B) and catheter ablation were performed using a high-resolution mapping system (Rhythmia; Boston Scientific). To avoid overlooking surviving low-voltage electrograms, a confidence mask with a cutoff value of 0.02 mV was applied during voltage mapping, as previously described for low-voltage mapping studies.^2^ A voltage map during AT (Figure 2A) demonstrated extensive absence of electrical activity in the sinus venarum and the right atrial septum, consistent with partial atrial standstill. The reconstructed 3-dimensional geometry indicated marked right atrial enlargement, with an estimated right atrial volume of approximately 335 mL. An activation map revealed that the right atrial muscular ridge, encompassing the arcuate ridge and the crista terminalis, served as a critical isthmus (Figures 2A to 2D, Video 1). Radiofrequency ablation targeting the right atrial muscular ridge resulted in cycle length prolongation and activation sequence change (Figures 3A to 3D). Entrainment pacing at the cavotricuspid isthmus (CTI) confirmed CTI-dependent AT, and subsequent CTI ablation successfully terminated the AT. No atrial tachyarrhythmias were inducible after burst pacing down to a cycle length of 200 ms.

## Outcome and Follow-Up

Severe bradycardia after ablation was managed with temporary pacing until permanent pacemaker implantation. Three days later, a permanent pacemaker was implanted, with a ventricular lead placed on the right ventricular septum. An atrial lead could not be implanted because of the absence of capture in the right atrium. Electrocardiography showed no discernible P waves, and transthoracic echocardiography demonstrated the absence of the A wave in the transmitral inflow, consistent with atrial standstill. The patient remained free of AT recurrence and cardiovascular events for 7 years while receiving direct oral anticoagulant therapy. Anticoagulation therapy was continued because atrial standstill itself was considered to carry a potential thromboembolic risk. Long-term follow-up included regular pacemaker interrogation and intermittent 12-lead electrocardiography. No recovery of sinus node function was observed during follow-up.

## Discussion

This case highlights the significance of the right atrial muscular ridge, including the arcuate ridge and crista terminalis, as a critical isthmus for macro-re-entrant AT in a patient with partial atrial standstill. The presence of anterior and posterior conduction barriers accentuated the anisotropic conduction within this structure. We propose the term “right atrial muscular ridge” because distinguishing the arcuate ridge and crista terminalis is challenging, given their continuous morphological and electrophysiological characteristics.

The anisotropic conduction properties may allow the right atrial muscular ridge to serve as a critical isthmus in the tachycardia circuit. The right atrial muscular ridge represents a morphological continuum extending from the crista terminalis through the arcuate ridge toward the interatrial septum and the Bachmann bundle,^3^ separating the right atrial appendage (RAA) from the sinus venarum. The crista terminalis is known to conduct rapidly in the longitudinal direction but slowly in the transverse direction.^4^ Furthermore, slowing of conduction or conduction block between the superior vena cava and the crista terminalis has been reported to be relatively common.^5^ In this case, partial atrial standstill involving the sinus venarum and the base of the RAA accentuated the anisotropic conduction properties of the right atrial muscular ridge, allowing it to serve as a critical isthmus. This finding suggests that electrically silent atrial tissue resulting from partial atrial standstill acts as a barrier to the AT circuit. Because of its robust structure and dense myofiber composition, the right atrial muscular ridge may be relatively resistant to electrical remodeling compared with other atrial tissues.

**Figure 4:**
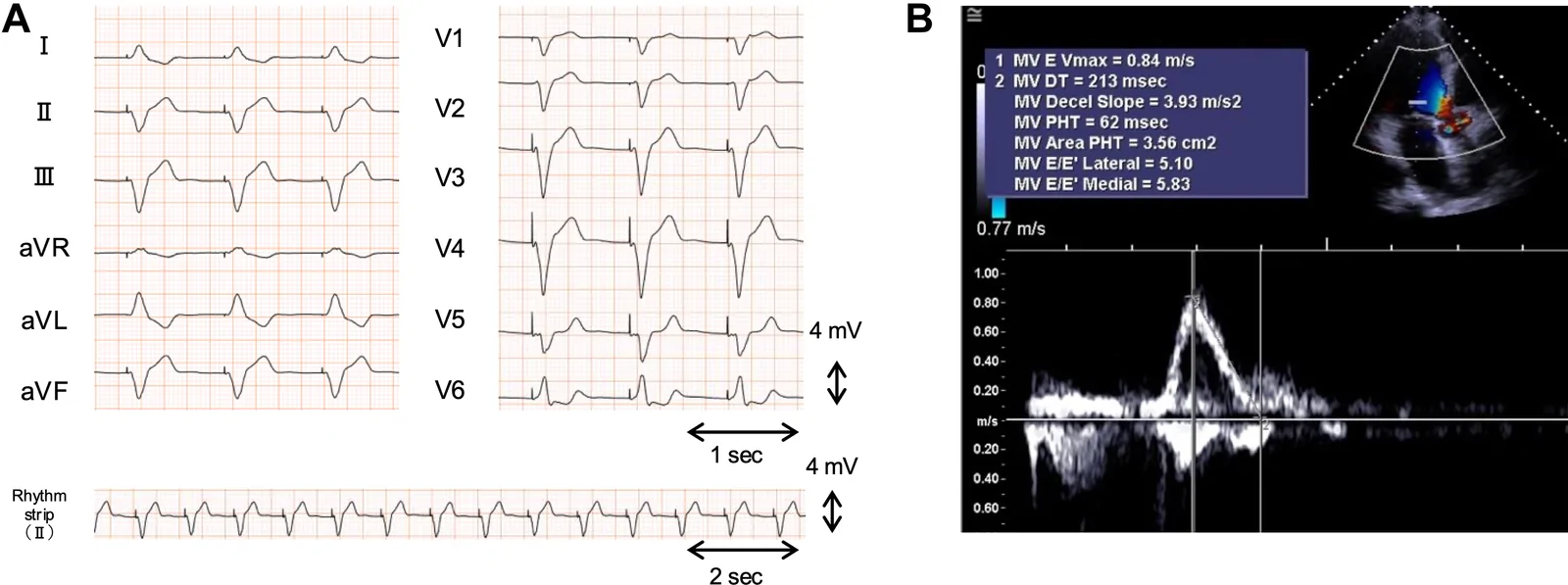
Postprocedural Transthoracic Echocardiography and Electrocardiogram (A). Transmitral inflow velocity waveform measured in the apical 4-chamber view, showing the absence of the A wave. (B). Electrocardiogram after pacemaker implantation, showing the absence of P wave.

We consider that the underlying substrate in this case represents idiopathic right atrial scarring, which served as the substrate for the observed macro-re-entrant AT circuit.^6^ After ablation, the patient fulfilled the diagnostic criteria^7^ for atrial standstill (Figure 4); however, based on preserved conduction before intervention, the preprocedural state was considered a partial form of atrial standstill. Although extensive electrically silent and low-voltage areas suggested preexisting sinus node dysfunction, the possibility of ablation-related sinus node injury could not be completely excluded because the ablation site was anatomically close to the sinus node region.^8^ In patients without a planned permanent pacemaker implantation, particular caution may therefore be warranted when performing ablation near this region.

Previous reports have demonstrated that patients who are later diagnosed with atrial standstill may exhibit atrial fibrillation or AT on earlier electrocardiograms,^1^ supporting the concept that atrial standstill represents a progressive disease spectrum rather than a static entity. Our case supports the concept that progressive atrial scarring may initially manifest as atrial tachyarrhythmias before evolving into total atrial standstill. Recognition of preserved atrial muscular ridges is therefore critical, as these structures may represent the only viable conduction channels and the most effective ablation targets in extensively scarred atria. This supports the concept that atrial standstill is not a static end-stage disease, but rather the terminal phenotype of a progressive atrial myopathic spectrum.

This case was classified as idiopathic partial atrial standstill, as no identifiable secondary causes^9^ were found. Persistent tachyarrhythmia itself may contribute to cardiac remodeling, whereas termination of tachycardia may prevent or delay progression. Therefore, rhythm control was initially attempted by ablation of the AT and pacemaker implantation after diagnosis of partial atrial standstill. Unfortunately, atrial lead implantation was impossible because atrial capture was not achieved. Although atrial pacing from the coronary sinus was possible during ablation, lead implantation into the coronary sinus was avoided owing to concerns regarding potential perforation. Further investigation is necessary to clarify the clinical significance of terminating tachyarrhythmia and atrial pacing in patients with partial atrial standstill.

## Conclusions

A continuum of the right atrial muscular ridge, including the arcuate ridge and the crista terminalis, can serve as a critical isthmus for macro-re-entrant AT in a patient with partial atrial standstill. Recognizing this infrequently reported structure and its anisotropic conduction properties is essential for electrophysiologists.

## Funding Support and Author Disclosures

This report received no specific grant from public, commercial, or not-for-profit funding agencies. Dr Kimura is an associate professor in the Department of Advanced Management of Cardiac Arrhythmias, an endowment department supported by Medtronic Japan Co, Ltd and Japan Lifeline Co, Ltd. He has also received speaker honoraria from Medtronic Japan Co, Ltd, Boston Scientific Japan Co., Ltd, Johnson & Johnson, and Toray. Dr Tomita received a research grant from Abbott Medical Japan LLC and is a concurrent professor of the Department of Advanced Management of Cardiac Arrhythmias. All other authors have reported that they have no relationships relevant to the contents of this paper to disclose.

## Appendix

Video 1 Propagation Map of the Tachycardia Propagation map viewed from the left anterior oblique (left panel) and right anterior oblique (right panel). The right atrial volume was estimated to be approximately 335 mL based on 3-dimensional chamber geometry reconstructed using the electroanatomic mapping system.
